# A novel smart navigation system for intramedullary nailing in orthopedic surgery

**DOI:** 10.1371/journal.pone.0174407

**Published:** 2017-04-17

**Authors:** Jaesuk Choi, Jihun Kim, Jae Youn Hwang, Minkyu Je, Jun-Young Kim, Shin-Yoon Kim

**Affiliations:** 1 School of Electrical Engineering, Korea Advanced Institute of Science and Technology, Daejeon, Korea; 2 Department of Information and Communication Engineering, Daegu Gyeongbuk Institute of Science and Technology, Daegu, Korea; 3 Department of Orthopedic Surgery, Kyungpook National University Hospital, Daegu, Korea; Georgia Regents University, UNITED STATES

## Abstract

This paper proposes a novel smart surgical navigation system for intramedullary nailing in orthopedic surgery. Using a handle-integrated laser guidance module, the system can target a drill insertion point onto skin, indicating an accurate target position to perpendicularly access an invisible distal hole. The proposed handle-integration-based fixation of the laser guidance module precisely defines the relative position of the module with respect to the distal hole. Consequently, unlike conventional systems, the proposed system can indicate the target insertion point without any help from bulky and costly external position-tracking equipment that is usually required for compensating disturbances generated by external impacts. After insertion, a correct drilling direction toward the distal hole is guided by real-time drilling angle measurement modules–one integrated with the nail handle and the other with the drill body. Each module contains a 9-axis inertial sensor and a Bluetooth communication device. These two modules work together to provide real-time drilling angle data, allowing calculation of the directional error toward the center of the distal hole in real time. The proposed system removes the need for fluoroscopy and provides a compact and cost-effective solution compared with conventional systems.

## Introduction

Intramedullary nailing has been considered one of most common and representative orthopedic surgical processes. This surgical procedure is carried out to minimize the rotation and displacement of a patient’s fractured bone. The nailing operation amounts to inserting a hollow nail into the medullary canal using a handle embedded into the nail, and then locking the holes of the nail using screws. However, since the nail is inserted into a bone, the surgeon does not see the locking area. Therefore, the locking procedure has been considered a challenging step in intramedullary nailing.

To tackle this challenge, a smart surgical navigation system is proposed in this work. The navigation system is widely used for various applications including marine [1] and aeronautic [2] navigation. The navigation is the process for guiding the movement of travelling objects such as ships and airplanes. The navigation system is supposed to indicate the correct position and direction for the object to travel so that the navigator can be guided accurately, even when the object is located in invisible sites. In the case of surgical navigation, the system provides surgeons with a guidance for the accurate control of surgical instrument, targeting a high-precision surgical operation to improve prognosis and minimize complication.

Commercial targeting-arm devices (TADs) have been proposed; these devices feature a jig to guide the proximal hole. However, no method to guide the distal hole exists. To solve this problem, a technique for guiding the distal hole is required in distal locking procedure. Most orthopedic surgeons use fluoroscopy to conduct the distal locking procedure, which exposes the patients as well as the surgeons to radiation. However, conventional radioscopy-based distal locking is not sufficiently accurate, because it is conducted by trial and error using two-dimensional (2D) fluoroscopic images, leading to the possibility of excessive errors. Furthermore, determining the distal hole using fluoroscopy requires more radiographic exposure compared with other orthopedic procedures. Because the human femur is slightly bent in the longitudinal direction, a nail is designed to have inclination and a curvature of several millimeters, to conform to the bone canal shape. The curvature and the inclination of such a twisted nail make it difficult for a surgeon to find the distal hole using one fluoroscopic image. Thus, determining the exact position of the distal hole requires acquiring and analyzing several fluoroscopic images.

In addition to the problem of inaccurate distal hole localization, the radiography-based approach has the following drawbacks: 1) It increases the operation time. A surgeon has to analyze a significant amount of visual data to align the direction of drilling toward the distal hole. Since in distal locking procedure, both targets (distal hole) and tools (drilling equipment and screws) are related to each other in the three-dimensional (3D) space, while the surgeon is performing the procedure based on 2D fluoroscopic images, a relatively large amount of radiographic data is required, thus increasing the operation time [3–5]. 2) It involves significant exposure to radiation, for both patients and surgical teams [3, 5]. Even though radioactive materials are denoted hazardous by the World Health Organization (WHO), they are still widely used in medical imaging. The reason for this is significant advantage in the resolution of imaging, compared with the radiation damage that is incurred.

Conventional methods for improving the nailing procedure can be classified into two categories: 1) methods that primarily improve the accuracy [4, 6, 7, 10, 11], and 2) those that minimize the use of radiation imaging [3, 5, 8, 9]. To improve the accuracy of the intramedullary nailing operation, Liao et al. proposed an autostereoscopic image overlay device combined with a laser guidance system [4]. This system can display 3D autostereoscopic images and reproduce motion parallax by superimposing the images of surgical anatomic structures onto the patient using integral videography. It specifies acquiring better accuracy by laser-guidance-based surgical instrument alignment method. Nakdhamabhorn et al. proposed a 4-DOF laser guidance robot which is highly accurate owing to fluoroscopic-image-based processing unit for calculating surgical path and optical tracking system for identifying position and orientation [6]. The robot guides the surgeon to perform highly accurate intramedullary nailing. Although these systems improve the operation accuracy, they still rely on radiographic images and the costs of configuring these systems are high [4, 6].

Many techniques and devices have been proposed to reduce radiation exposure associated with 2D radiography [3, 5, 8, 9]. Magnetic-field-sensing-based techniques have been proposed for determining the distal hole location without using radiography [3, 8]. Lee et al. proposed a remote guiding device that finds the screw hole position for drilling and the direction for screw locking. This system consists of three magnetic pins and electrical conductive board. By moving the device where the magnetic pins are mounted to align with the magnet in the nail, the drilling position and screwing direction can be guided [3]. The drawback of this system is that the magnetic field can be distorted when the conductor is adjacent to the nail. In addition, this system requires custom manufacture of nails with a permanent magnet inside. Chu et al. proposed a nail-embedded endo-trans illuminating system [5], which emits light while a nail is positioned in the bone marrow. This system uses a light-emitting diode (LED) that emits light with wavelength in the visible range (700–1000 nm). The light projected onto the bone surface reveals the position of the screw hole, and allows the subsequent drilling and placing of the locking screw without the need of using radiography. However, even though the operator can see the light emitted from inside of the bone marrow, the guiding light is scattered owing to tissue reflection, requiring an additional optical device. In addition, the proposed system is not convenient to use. After locating the nail into the bone marrow, the user has to disassemble the nail handle and insert the optical structure including the LED inside the nail. Both of the proposed systems require specially manufactured nails, thus increase the cost.

To sum up, the guiding systems reported thus far have significant limitations (Table 1). The systems failed to achieve elimination of radiation exposure, operational accuracy, cost effectiveness, or user convenience. In this paper, we present a novel system that includes handle-integrated line-laser markers and instrument-integrated smart module. This system is low-cost and offers accurate distal locking method in intramedullary nailing, without any radiation exposure. Using this system, screw insertion points can be accurately localized on skin, and a surgeon can verify the real-time angle of surgical drilling, which corresponds to the direction of the drill bit toward the distal hole. The results of this study demonstrate that the proposed system is easy to use, precise, low-cost, and radiation-free, compared with previously proposed systems.

**Table 1:** Comparison with other systems reported previously.

	Liao et al. [4]	Nakdhamabhorn et al. [6]	Lee et al. [3]	Chu et al. [5]	This work
**Method**	Optical tracking based on fluoroscopic images	Optical tracking based on fluoroscopic images	Electromagnetic sensing	Light emission from inside of the bone marrow	Handle-integrated laser projection & inertial sensing
**Accuracy**	High	High	Low	Medium	High
**Radiation exposure**	Required	Required	None	None	None
**User convenience**	Poor	Poor	Medium	Medium	Good
**Cost**	High	High	Medium	Low	Low
**Key advantage**	High-accuracy		Low-radiation		Both

## Materials and methods

### Handle-integrated line-laser markers

In this paper, we propose a handle-integrated laser guidance module for localizing the screw insertion point. Conventionally, several laser guidance systems have used dot-pointed laser modules [9]. However, in a dot-pointed laser, the output position of the laser beam varies in accordance with the height of a medium. Thus, if a dot-pointed laser is simply adopted for intramedullary nailing, the screw insertion point can change according to the thickness of patient’s soft tissue.

**Fig 1:**
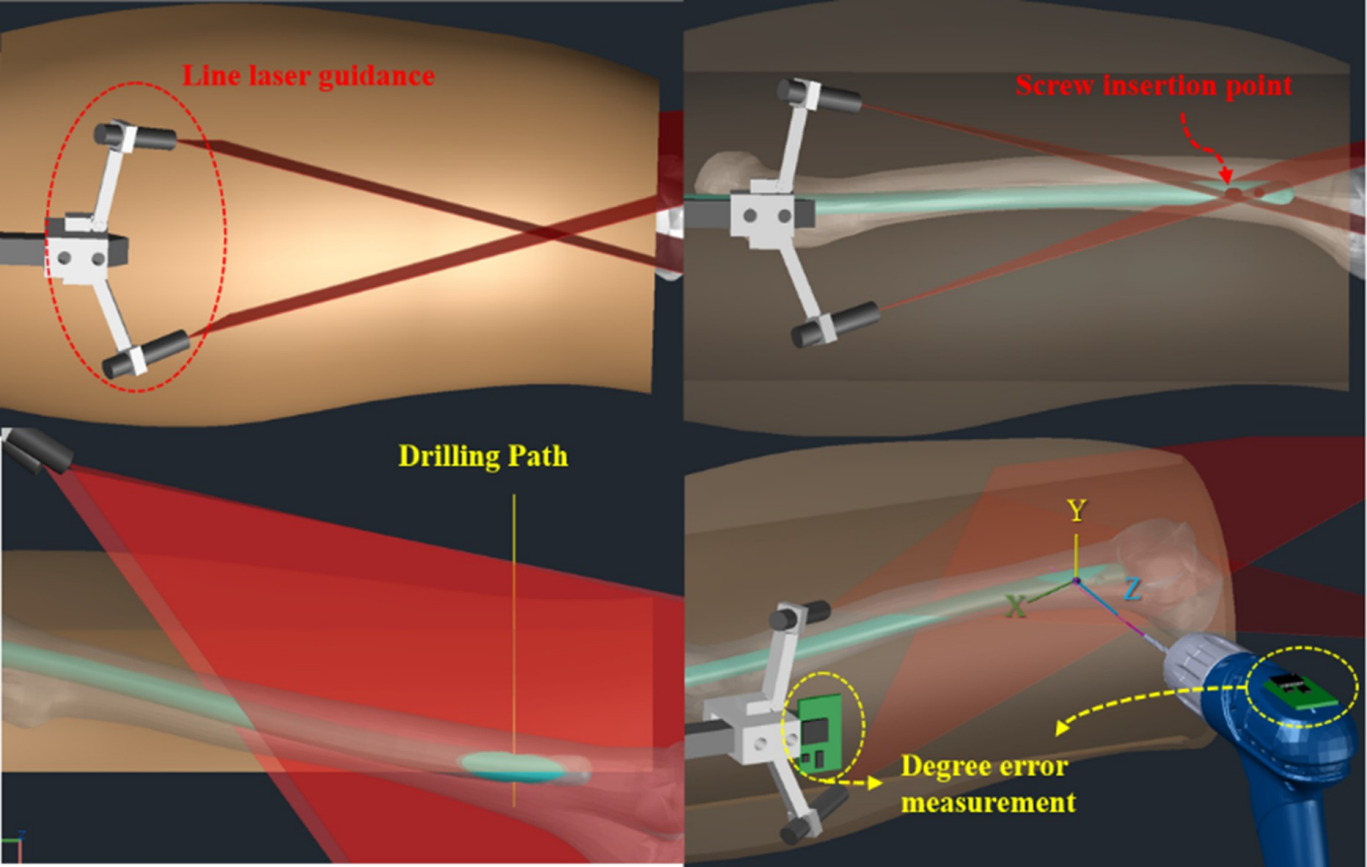
The proposed navigation system. Schematic view of screw insertion point localization and real-time measurement of the drilling angle.

In our system, we used the output beam planes of two line lasers which had a line of intersection [7]. As shown in Fig 1, the laser guiding module includes two line lasers, projecting two individual lines on the skin surface. When the lasers emit light toward the distal hole, the line of intersection of the corresponding beam planes is normal to the plane of the distal hole. When this normal line crosses the patient’s skin, an intersection is formed, indicating the precise insertion point of a locking screw, from which the target distal hole can be perpendicularly accessed, regardless of the tissue thickness. In addition, the proposed laser guidance module is coupled to a handle that is used when inserting the nail. The advantage of coupling the laser guidance module to the handle structure will be discussed later.

**Fig 2:**
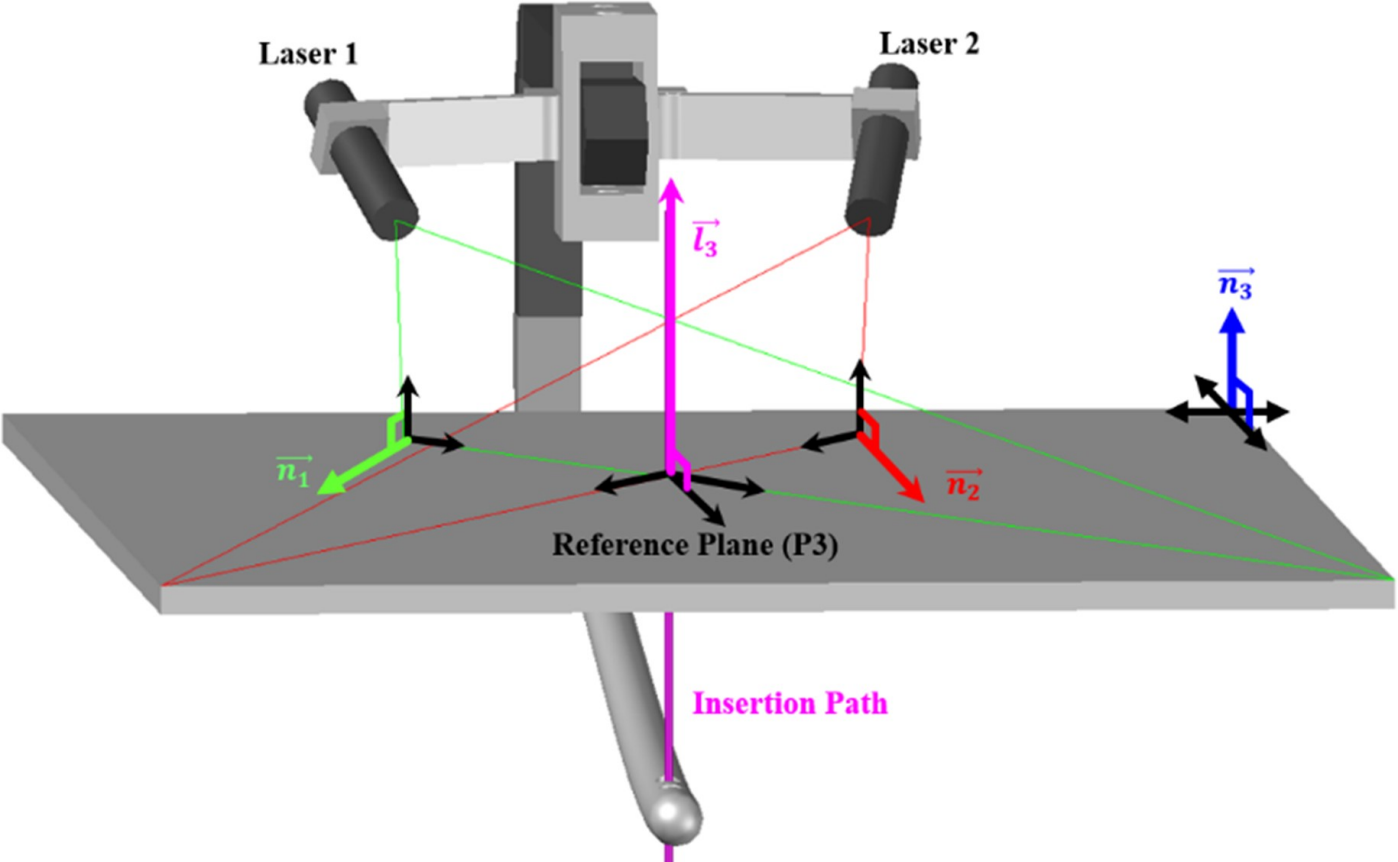
The principle of the proposed laser guidance system. The planes of the beams emitted by the two lasers are perpendicular to the reference plane, and the distal hole is located on the intersection line between the planes of the two laser beams.

To mathematically interpret the principle of the laser guidance system in Fig 2, we first assume that the reference plane is located on the patient’s skin into which the screw is inserted, by translating the distal hole plane. Then, the two laser markers are adjusted in such a way that the beam planes (P1, P2) formed by the light beams emitted toward the distal hole are normal to the reference plane.

$$\vec{n_{1}}∙\vec{n_{3}}=0$$ (1)

$$\vec{n_{2}}∙\vec{n_{3}}=0$$ (2)

$$\vec{n_{1}}=Normal\;vector\;of\;plane\;1$$

$$\vec{n_{2}}=Normal\;vector\;of\;plane\;2$$

$$\vec{n_{3}}=Normal\;vector\;of\;the\;reference\;plane$$

The intersection of the two laser beam planes ($\vec{l_{3}}$) is where the insertion point is localized; the intersection line is always normal to the distal hole.

$$\vec{n_{3}}\times \vec{l_{3}}=(\vec{n_{1}}\times \vec{n_{2}})\times \vec{l_{3}}=0$$ (3)

$$\vec{l_{3}}=Vector\;of\;crossing\;beam\;planes$$

Using the set of locational relations above, we developed a low-cost drill insertion guiding module for distal locking.

However, line-laser-marker-based systems for intramedullary nailing have a practical drawback; these systems are sensitive to possible surgery site displacement during operation. Conventionally, laser guiding modules are positioned with no fixed relation to the location and orientation of the nail, and any disturbance of the patient’s femur owing to an external physical impact displaces the surgical site, yielding localization errors. For example, even when the reference plane (surgical site) is tilted, projection angles do not change along with the reference plane because the laser guidance device is separated from the nail and handle. Thus, after the displacement, the normal vectors of the laser beam planes are not perpendicular to the reference plane anymore. Let us denote the normal vectors of the three planes by $\vec{n_{1}}$, $\vec{n_{2}}$, and $\vec{n_{3}}$.

$$\vec{n_{1}}=(a_{1},b_{1},c_{1})$$ (4)

$$\vec{n_{2}}=(a_{2},b_{2},c_{2})$$ (5)

$$\vec{n_{3}}=(a_{3},b_{3},c_{3})$$ (6)

If the distal hole plane, and hence the reference plane are tilted by θ°, the normal vector of the reference plane $\vec{n_{3}}$ can be expressed as follows.

$$\vec{n_{3}^{\prime }}=(a_{3},\;b_{3}cos\theta -b_{3}sin\theta ,\;c_{3})$$ (7)

Then, the intersection line of the beam planes ($\vec{l_{3}}$) is not parallel to the normal vector of the reference plane.

$$\vec{n_{3}^{\prime }}\times \vec{l_{3}}\neq 0$$ (8)

Thus, the point of intersection of the laser-beam planes does not indicate the distal hole location, i.e. a guiding error occurs.

To solve this problem, previously proposed laser marker based systems have used additional optical devices and data processing devices for calculating proper lasers’ projection angles [4, 6]. Additional devices are used for tracking the operating site’s movement and for calculating the resulting errors, so that the lasers’ projection angles can be properly controlled. It makes the system more expensive and obstructive. In [4], the direction of the surgical instruments toward the target is indicated by superimposing the patient and the surgical instruments using the integral videography image overlay device equipped with the laser guidance device. In addition, a separate optical tracking system was used for the calibration of the laser guidance. However, since this system uses a motor driving module to control the laser, an image overlay device for visualization, and an optical tracking device for calibration, the production cost of the system is high and the pre-operation step such as image registration of patient data is required.

On the other hand, our system uses a robust fixation mechanism with mechanically integrated handle and laser-guidance module. As a result, the laser guidance module is automatically adjusted, offsetting the surgical site displacement. Thus, the module always locates a correct insertion point, which is perpendicular to the center of the distal hole. When comparing the accuracy of our system (Distance error: 2.16 mm, Orientation error: 2.25°) with the accuracy reported in [4] (Distance error: 2.48 mm, Orientation error: 2.96°), there is no significant difference in the system performance, while our system has much simpler and cost-effective configuration as well as easier operation process.

For properly localizing the insertion point, it is also important to compensate the axial mismatch between the distal hole and the handle. Because the nail is driven into the bone, the nail has a slightly bent curvature to conform to the patient’s anatomy; thus, the axis of the handle is tilted relative to the axis of the distal hole. This axial mismatch should be accounted for when calculating the lasers’ emission angles.

**Fig 3:**
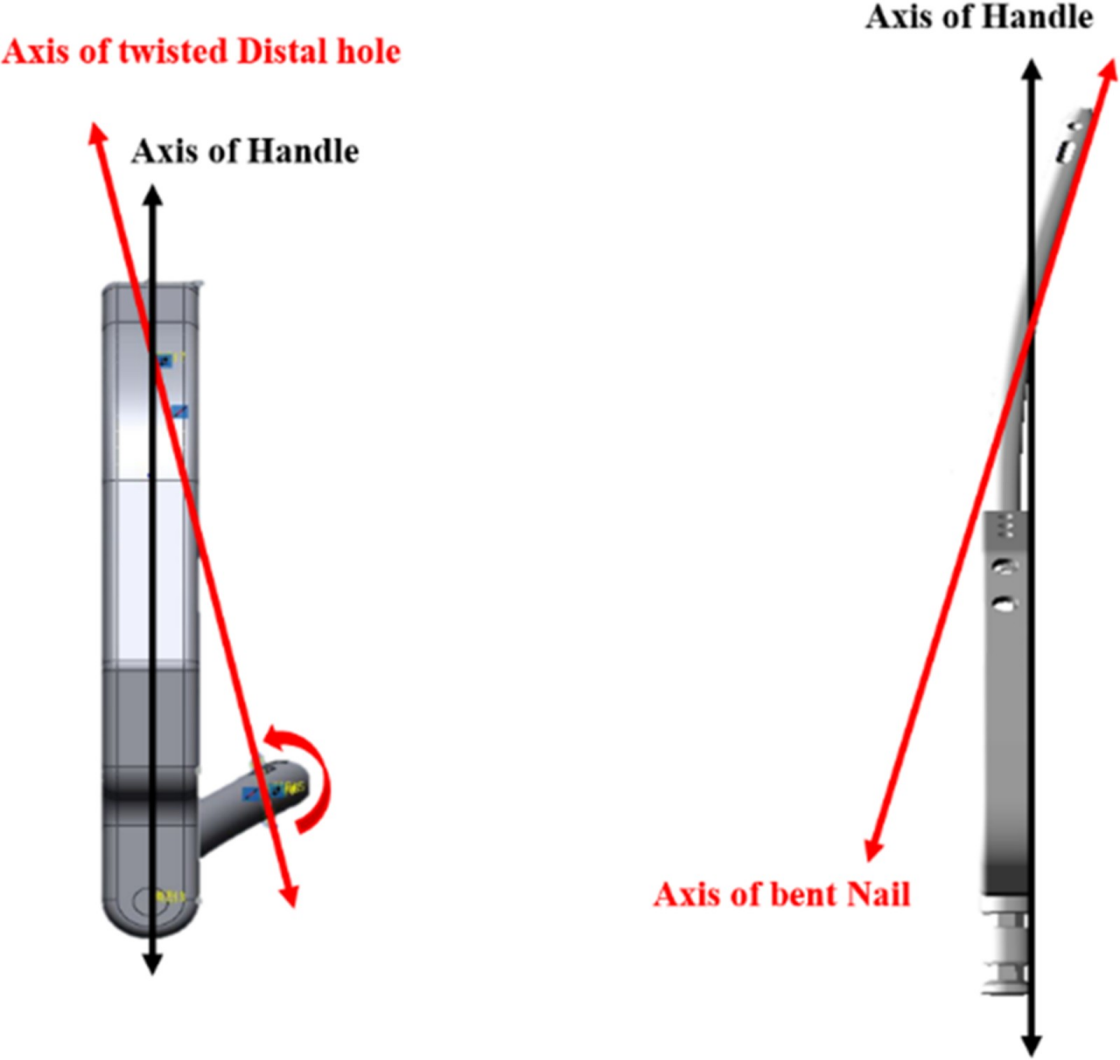
The 3D models of the handle and nail. They were designed for measuring the mismatch between the axis and the insertion angle.

**Fig 4:**
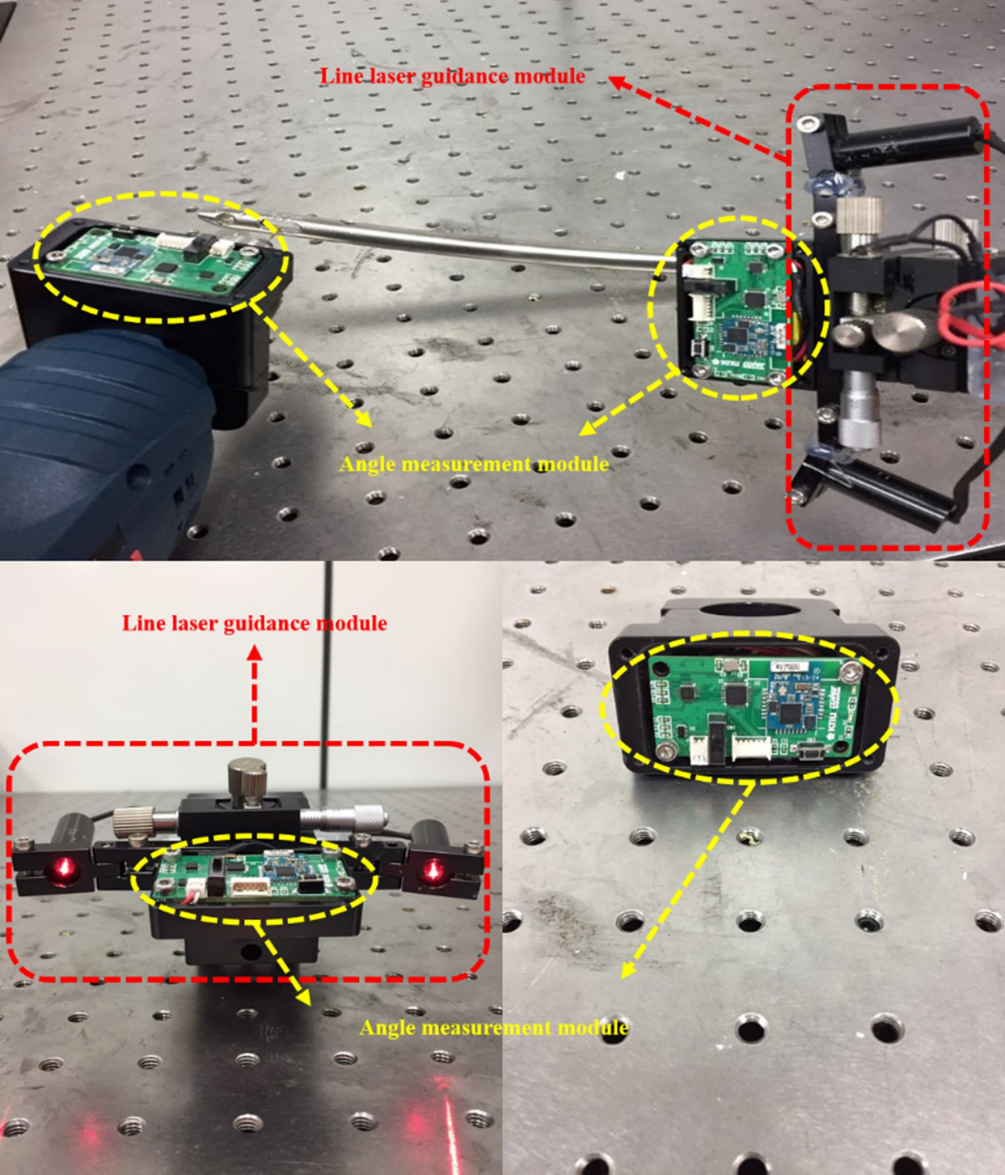
The designed modules of the navigation system. Photographs of the line-laser guidance module and real-time angle measurement module.

As shown in Fig 3, to resolve these issues, accurate 3D models of the nail and handle structures were constructed. By compensating the lasers’ emission angles according to the measured tilt and bending angle (axis mismatch), the laser mounting structure prevented the effects of axial mismatch from occurring. To control the angle and the direction of laser guiding, as shown in Fig 4, the module structure was designed to be direction-controllable. In the present work, the module was hand-controlled, but automatic control will be introduced in the future. The nails are classified into several types, depending on the size of the patient’s femur. However, the present system employs only one nail type, because the present study focuses on validating the proposed laser guidance module.

**Fig 5:**
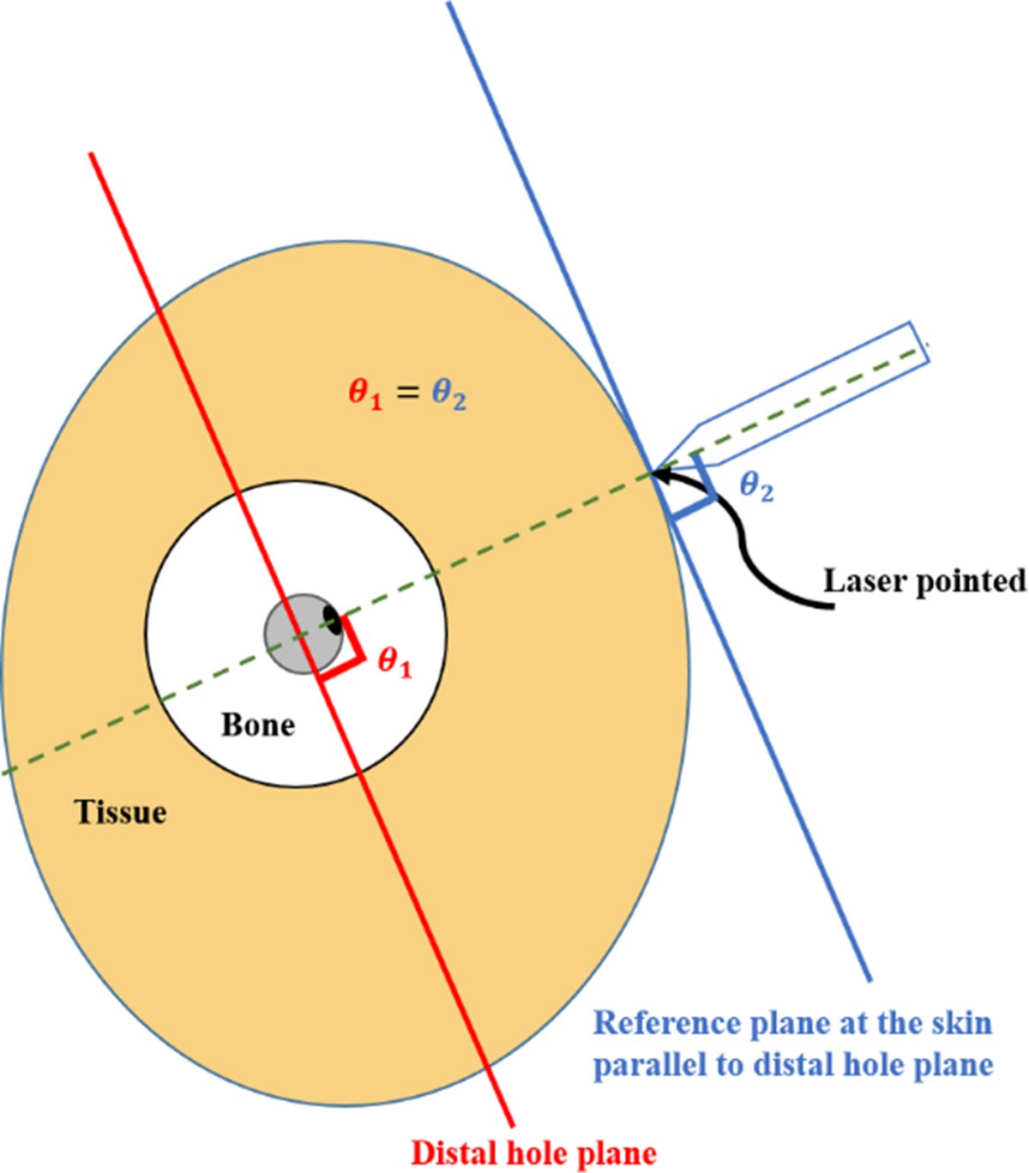
Diagram of cross-sectional view in distal thigh. It shows the geometrical relation between the drill insertion point guided by the system and the distal hole of the nail.

In our system, the laser markers easily localize the precise insertion point using the proposed handle-integrated structure. Because the system contains a robust integration mechanism and uses accurate geometric calculations, the projection angles of laser markers are automatically controlled along with the nail movement, alleviating the need to use additional devices for tracking and compensation. The proposed structure ensures that the laser guided point is always located on the line perpendicular to the center of the distal hole, as shown in Fig 5. The operating surgeon is conveniently guided toward the correct point into which the surgical instrument is to be inserted. The proposed system is cost-efficient.

### Drilling direction guidance smart module

In intramedullary nailing, after guiding toward the accurate location of the insertion point, screw drilling is performed as a next step toward successful distal locking. It is important not only to ensure precise localization but also to provide an accurate drilling direction guidance, because inaccuracy in screw drilling can result in a screw crash and unstable distal locking. Conventionally, surgeons verify the screwing direction while drilling using fluoroscopic imaging. In addition to being based on radiography, this approach is time-consuming.

Implementing real-time navigation for a direction guidance system is a challenge, and many approaches have been proposed for solving this issue [3, 8]. However, most of these approaches did not find general acceptance, owing to their high associated cost. Employing a conventional navigation system incurs an excessively higher cost in performing nailing procedures. Given the cost constraint, we focused on implementing a low-cost and precise drilling direction guidance system.

As shown in Fig 2, our proposed system relies on the laser guidance module, which ensures that the insertion point is always located on the line that is perpendicular to the plane of the distal hole. This allows to successfully perform the surgery, as long as the drill is kept heading in perpendicular direction. In our approach, the drilling angle is measured using inertial sensors, for allowing the surgeon to ensure that the drilling direction is perpendicular to the plane of the distal hole. Therefore, our system does not require fluoroscopic imaging for real-time validation of drilling direction. However, the drilling angle should be adjusted in real time, to offset any potential displacement of the surgical site. Thus, the drilling direction with respect to the plane of the handle that is embedded into the nail should be measured in real time. To achieve this, we employ two angle measurement modules–one integrated with the body of the drill and the other with the handle of the nail.

For implementing each angle measurement module, a 9-axis inertial sensor chip, a Bluetooth communication module, and a microprocessor unit are used. Each module measures the tilt angle in real time and transmits the measured data to a host computer having a display panel. The host computer then processes the data received from two modules to calculate the drilling direction with respect to the plane of the handle and displays the results. By observing the information on the display panel, the surgeon can perform real-time verification of the drill’s current position and the tilt angle of the drill bit relative to the plane of the distal hole, so that the actual drilling path can be aligned with the target path, as in Fig 1.

**Fig 6:**
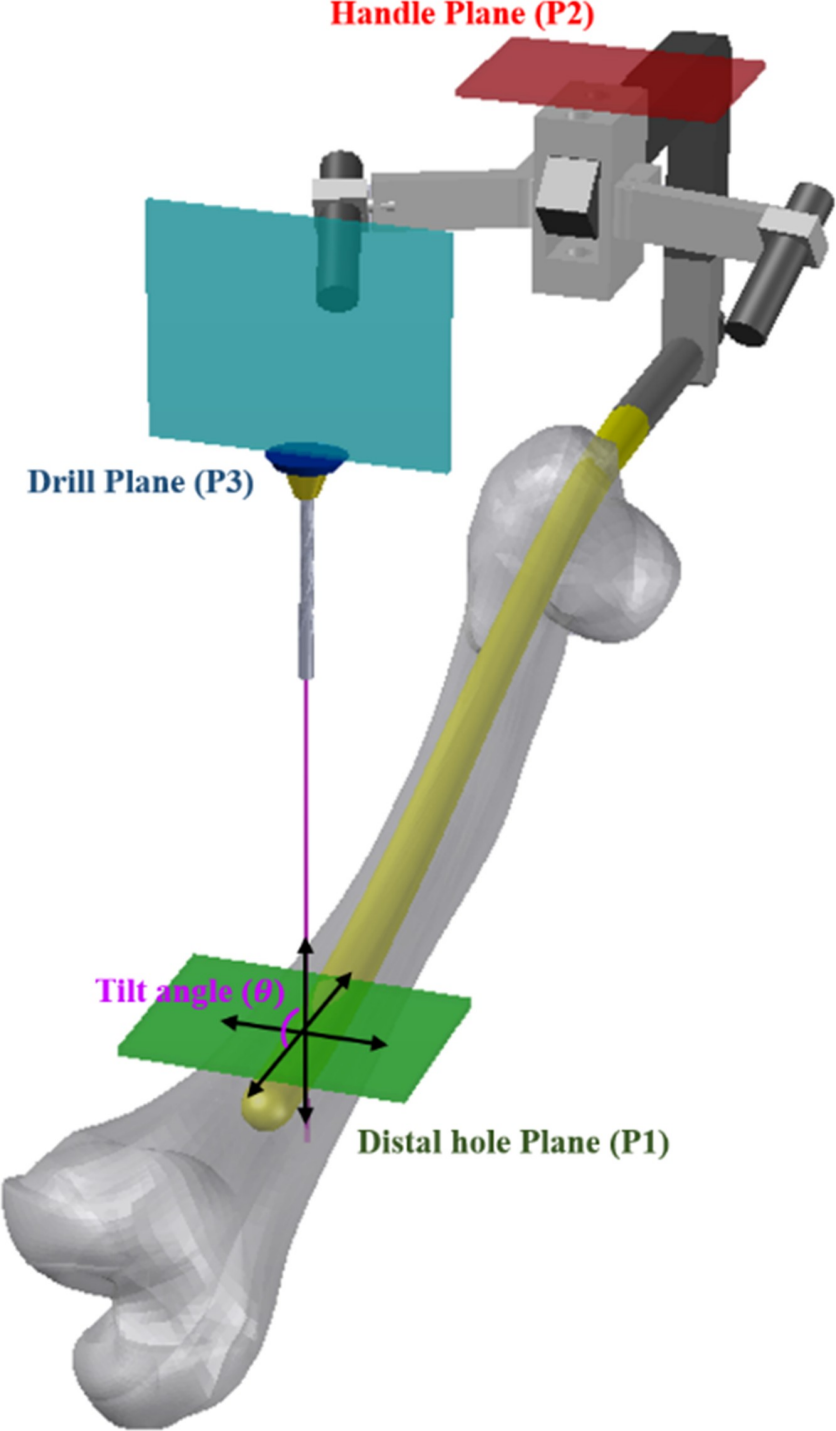
Alignment of different planes for ensuring perpendicular insertion of the screw.

**Fig 7:**
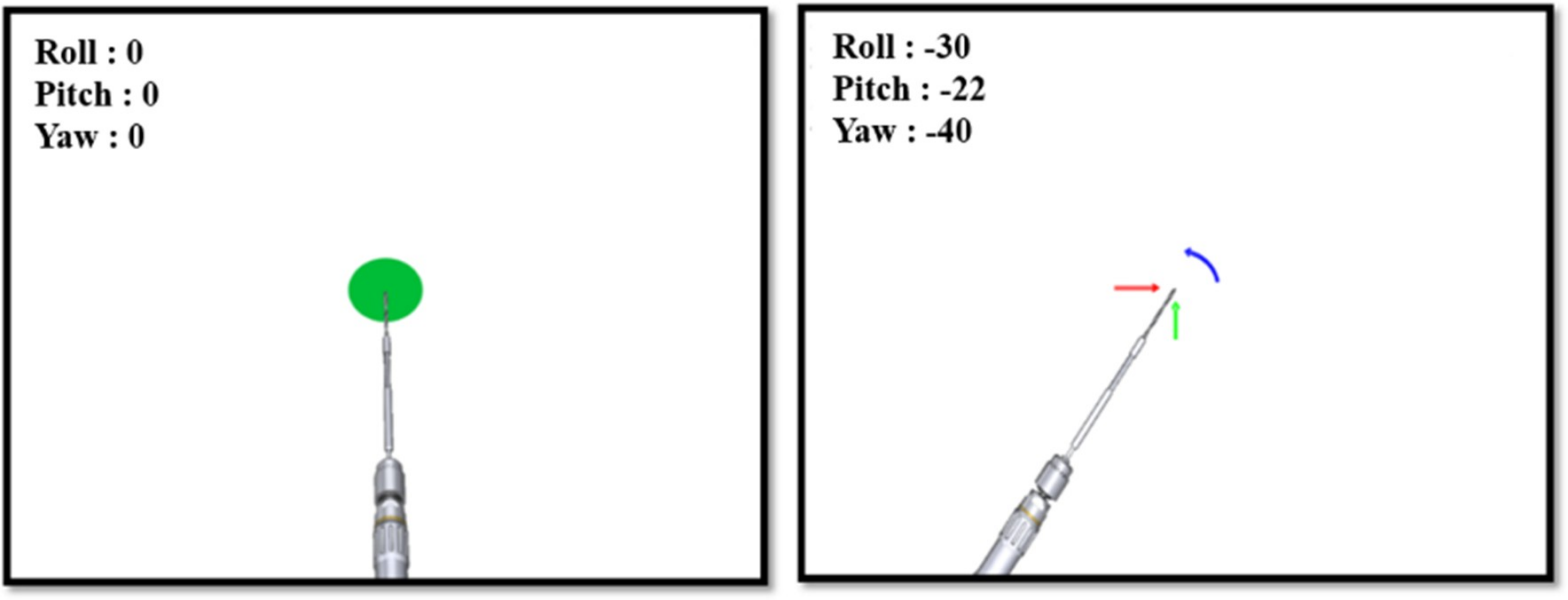
Real-time visual feedback on the drill’s path.

The method of drilling direction alignment for screw drilling is shown in Fig 6. As the plane of the handle (P2) and the plane of the distal hole (P1) are interconnected structurally, the angles between the planes are always the same. Because successful distal locking requires the drill to be inserted perpendicularly, the drilling angle with respect to the distal hole plane should be measured in real time. When the drilling angle is tilted relative to the distal hole plane (P1), an error is registered by the integrated angle measurement modules. Then, as shown in Fig 7, the measured error in the form of Euler angles (yaw, pitch, and roll rotations) is visually fed back to the surgeon, along with the information on the correct drilling path. When the drilling direction is correctly aligned with the direction toward the distal hole, a green circle is displayed at the tip of the drill bit. If the drilling angle is not correct, the measured real-time error value is displayed together with the arrows indicating the direction of required adjustment.

### Design of electronic circuits

**Fig 8:**
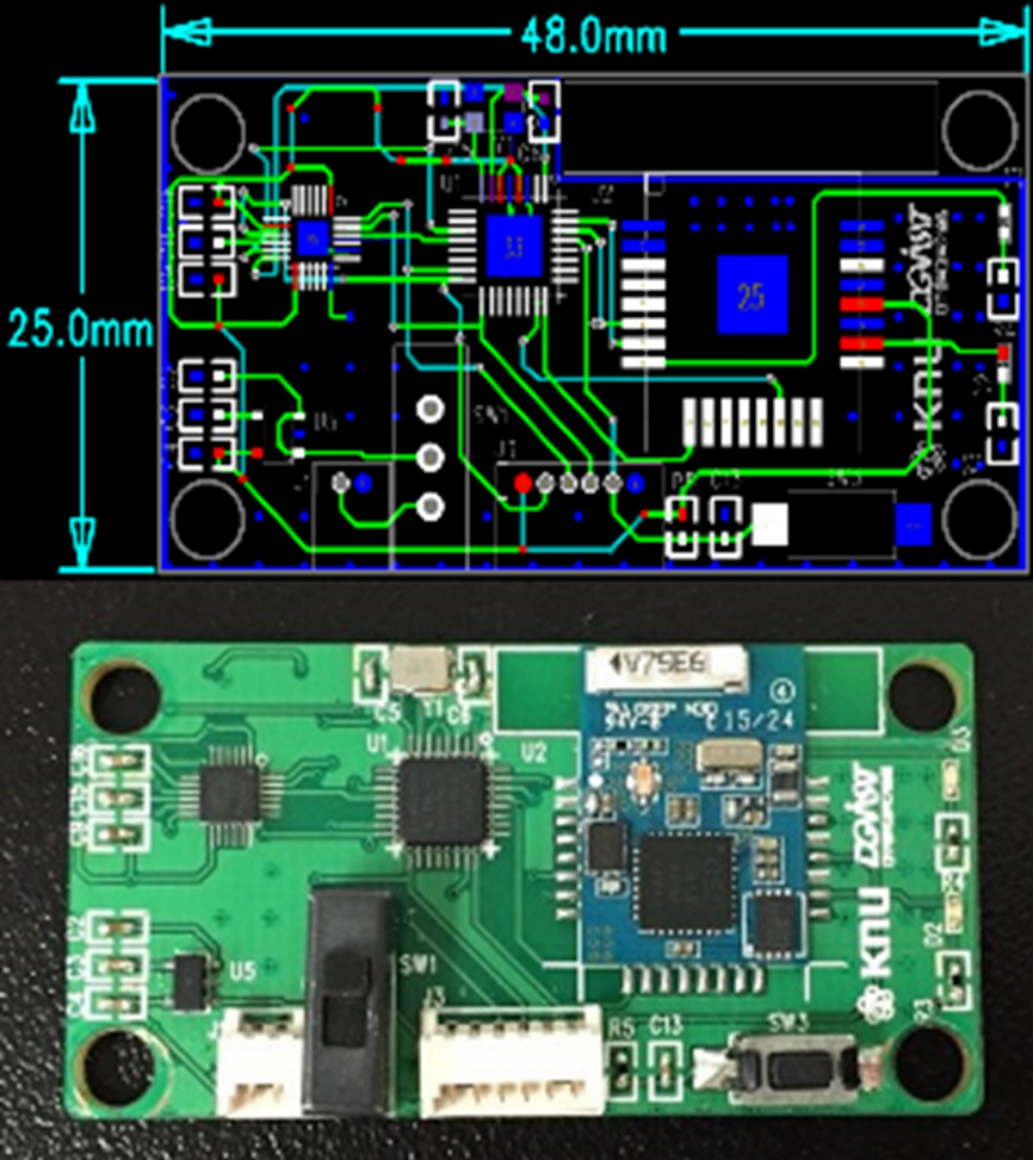
Electronic circuits implemented on the printed circuit board (PCB) for the angle measurement module.

The circuit module (Fig 8) of the provided drilling angle guidance system consists of a sensor circuit for real-time angle measurements, a communication circuit for communicating with the host computer, and a power management circuit for providing proper power to all circuit blocks. In the sensor circuit, a 9-axis inertial sensor (MPU-9250), containing a gyroscope, an accelerometer, and a magnetometer, is used for measuring displacement data in real time. In addition, the system features a micro-processor (ATmega-168) module for converting raw displacement data into degree data, using a custom algorithm. A Bluetooth communication module (BOT-CLE110) is used in the communication circuit to transmit real-time angle information measured from the handle and drill integrated modules. The transmitted information is processed in the host computer to provide the real-time visualization on the display panel. The Galaxy Tab (Samsung Electronics Co., Suwon, Korea) is used in this work.

When choosing the electronic circuit chip for our system, we considered its size, power consumption, and temperature limitation. For surgeons not to feel obstructive, the module size should be as small as possible, and the operating time should be long. The circuit board that was implemented in this study was 48 × 25 mm^2^ as shown in Fig 8, and starting with a fully charged battery, the operating time was about 20 hours. Importantly, because medical devices are sterilized pre-operation, the circuit should withstand sterilization temperature. Medical devices are usually sterilized using the autoclave method, which requires the temperature of 120°C, which can be too high for the circuit to withstand. To be on the safe side, we therefore propose to use a different sterilization method, utilizing the EtO (ethylene oxide) gas. The EtO gas sterilization method does not require the temperature higher than 60°C.

### Drill vibration effect

Due to the vibration of a drill, sensing the real-time tilt angle of the drill is distorted while the drill is being inserted. The drilling noise is determined based on the location of motor and gear. Especially the gear driving part in the drill accounts for up to 60% of overall vibration. Thus, the noise that is caused by the drilling vibration should be accounted for. We positioned the angle measurement module apart from the drill-involved motor and gear, which structurally are the most intensive vibrating parts. Hence, the effect of the drill’s vibration noise was alleviated.

### Estimation of orientation using a complementary filter based on Madgwick’s algorithm

To accurately estimate 3D orientations of a drill and the distal hole of a nail with respect to the direction of gravity and the magnetic field of Earth, using inertial and magnetic sensor units, we employed an orientation algorithm based on the gradient descent method proposed by Madgwick et al [12]. It has advantages such as a low computational load and a low sampling frequency, compared with conventional 3D orientation estimation algorithms [13, 14]. In the estimation of the orientation of a target object using a magnetic sensor, magnetic distortion inevitably occurs, owing to the effects of hard and soft irons. To mitigate this magnetic distortion, a reference direction for the magnetic field of Earth was predefined in previously proposed methods [15, 16]. In contrast, Madgwick’s algorithm does not require to predefine the reference direction, since it can compensate magnetic distortions [12].

**Fig 9:**
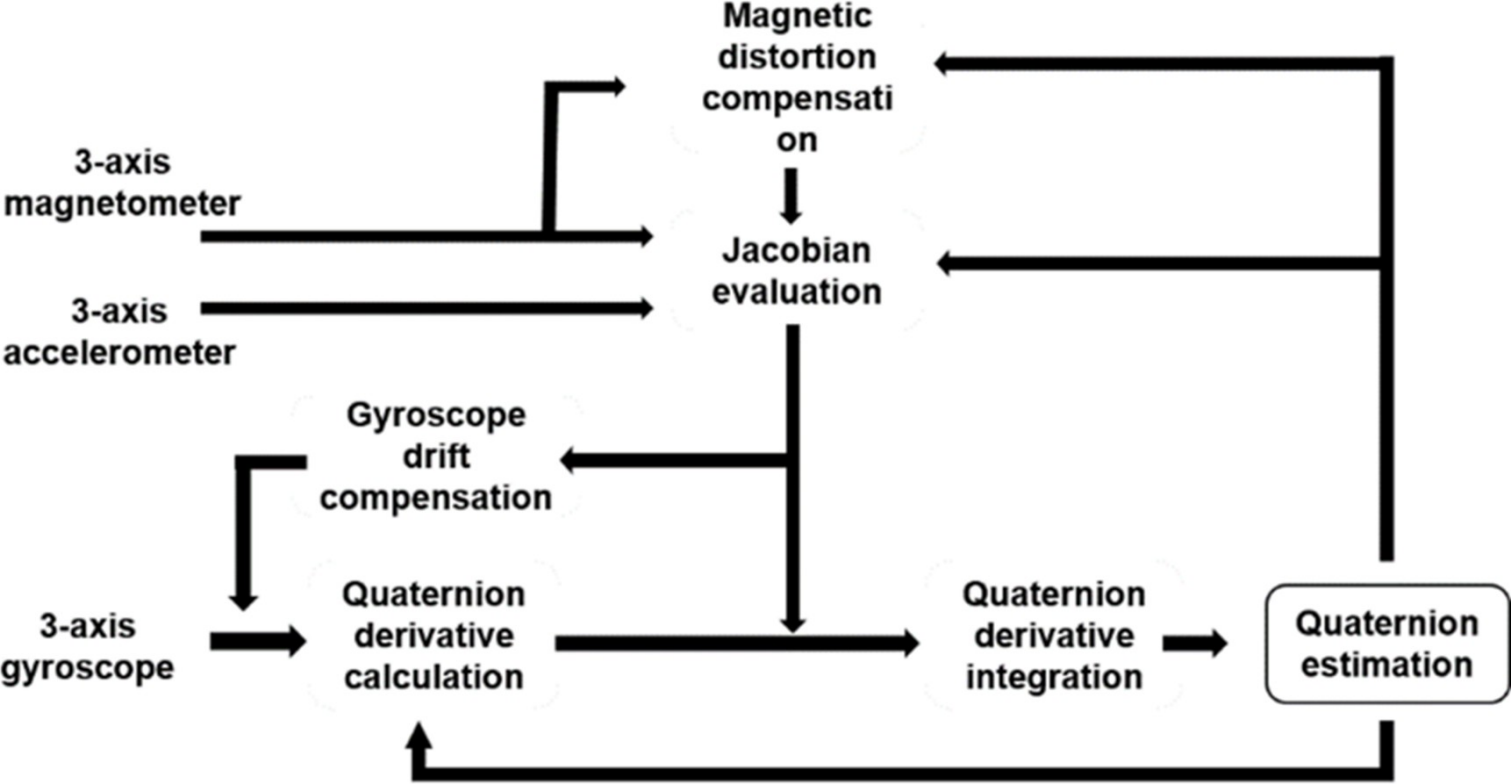
Block diagram of the 3D orientation estimation method based on Madgwick’s algorithm.

Fig 9 illustrates the 3D orientation estimation method based on Madgwick’s algorithm for real-time tracking of orientations of a drill and the distal hole of a nail. Here, we used an inertial sensor unit, MPU-9250 (Invensense, USA), which is composed of a 3-axis accelerometer, a 3-axis gyroscope, and a 3-axis magnetometer for 3D orientation estimation of a drill and the distal hole of a nail. Using the sensor unit, the acceleration and angular rates of a moving target object and the magnetic field of Earth are measured. Based on this information, the attitude and the heading direction of the drill and the distal hole of the nail with respect to the direction of gravity and the magnetic field of Earth can be estimated. In the algorithm, after calculating the quaternion derivatives of the measured 3-axis angular rate values, the derivatives are integrated to obtain the orientation of the sensor frame with respect to the reference frame of Earth. The gyroscope bias drift error that occurs in the quaternion derivative integration stage over time, temperature, and motion, is compensated by orientation filters based on the integral feedback of the error with an appropriate gain. The filter gain, which is expressed by the magnitude of a quaternion derivative of an estimated rate of the gyroscope bias drift, determines the rate of convergence for removing the drift errors.

## Results

**Fig 10:**
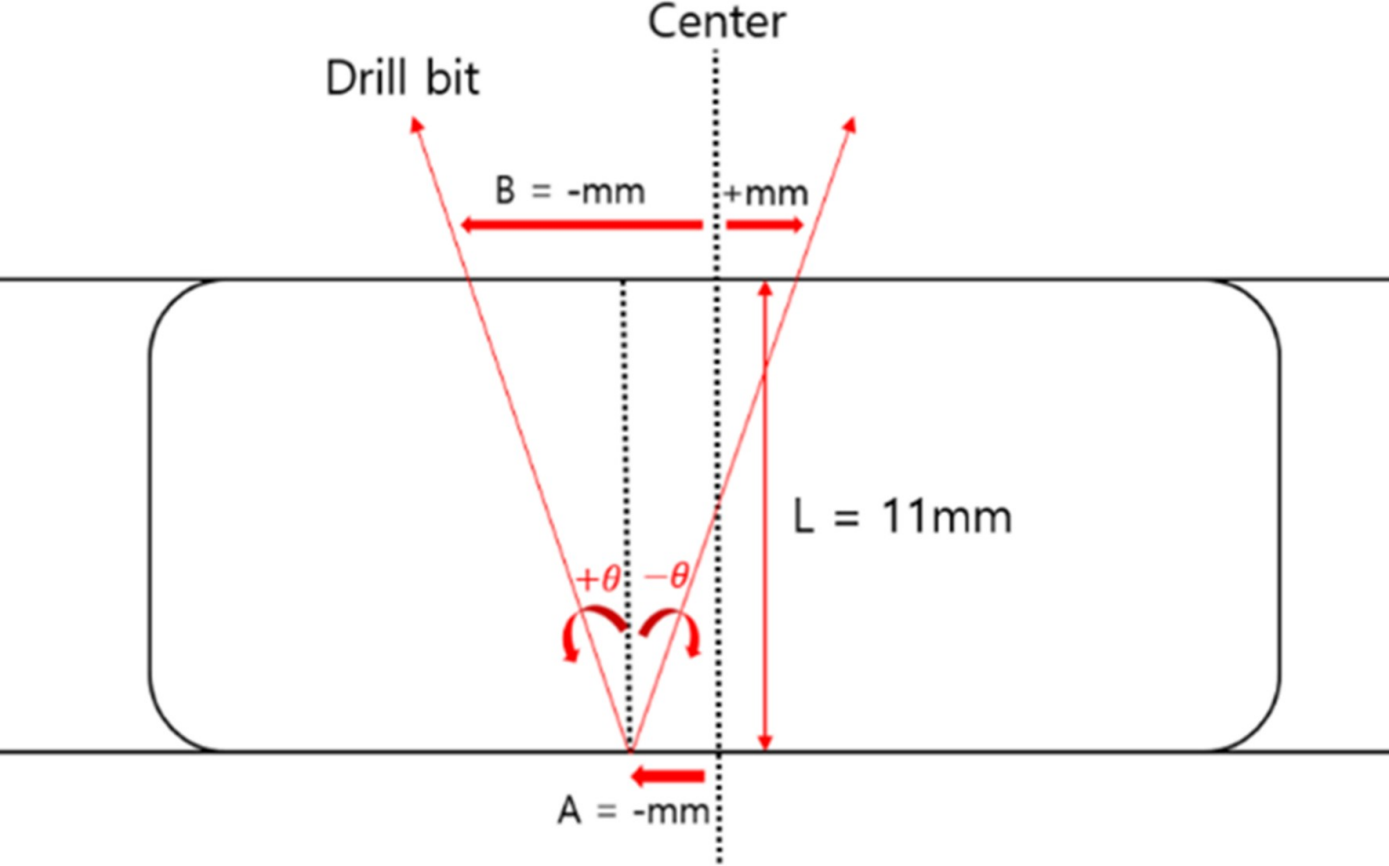
Schematic cross-sectional view of distal hole in nail. The black-dotted line in the center represents the desired insertion path. The red arrow line represents an incorrect path of the drill bit.

Fig 10 illustrates the distal hole of a nail in the lateral view. L is the depth of the distal hole of the nail. Performance of the smart navigation system for intramedullary nailing in an orthopedic operation was evaluated by measuring the following parameters: (1) the incidence angle (θ) of a drill bit; (2) the distance between the center line of the distal hole of a nail and the insertion point of the drill bit (A); (3) the distance between the center line of the distal hole of the nail and the exit point of the drill bit (B).

The incidence angle (θ) of a drill bit represents the accuracy in the orientation estimation of the drill and the distal hole of a nail, obtained using the 3D orientation estimation method. The distance A between the center line and the insertion point represents the allowance error, indicating the extent to which the screw insertion point is perpendicular to the center point of the distal hole of the nail. The distance B between the center line and the exit point indicates the accuracy in the tracking the drill orientation in real time.

**Fig 11:**
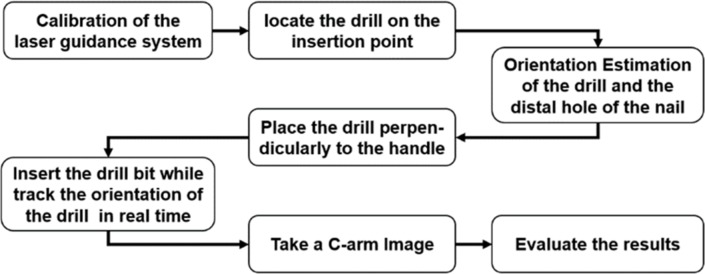
Experimental procedures for validating the developed navigation system.

Fig 11 demonstrates the experimental procedures for validating our developed system: 1) the laser guidance system is calibrated manually for indicating the screw insertion point perpendicular to the distal hole of a nail before intramedullary nailing is started; 2) the tip of the drill bit is located at the insertion point indicated by line lasers; 3) the orientations of the drill bit and the distal hole of the nail are estimated simultaneously and the orientation values are displayed on a display panel using an Android application. Therefore, an operator can monitor the orientations of the drill bit with respect to the distal hole of the nail in real time; 4) the drill bit is then placed perpendicularly to the distal hole of the nail using the orientation information; 5) the drill bit is inserted into the distal hole of the nail while the orientation of the drill bit is tracked in real time; 6) a C-arm image is acquired to evaluate whether the drill bit is properly inserted into the distal hole of the nail.

**Fig 12:**
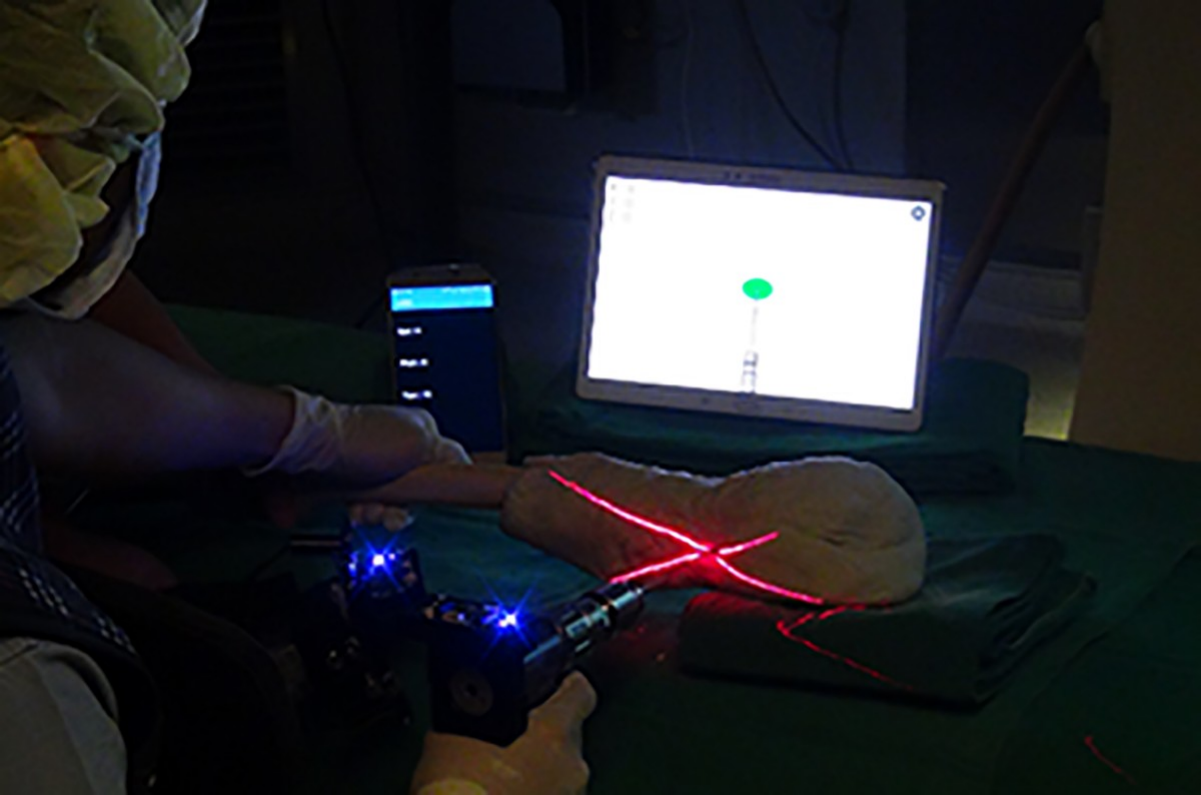
In-vitro study using the laser guidance system with the orientation estimation system integrated in the drill and the handle.

**Fig 13:**
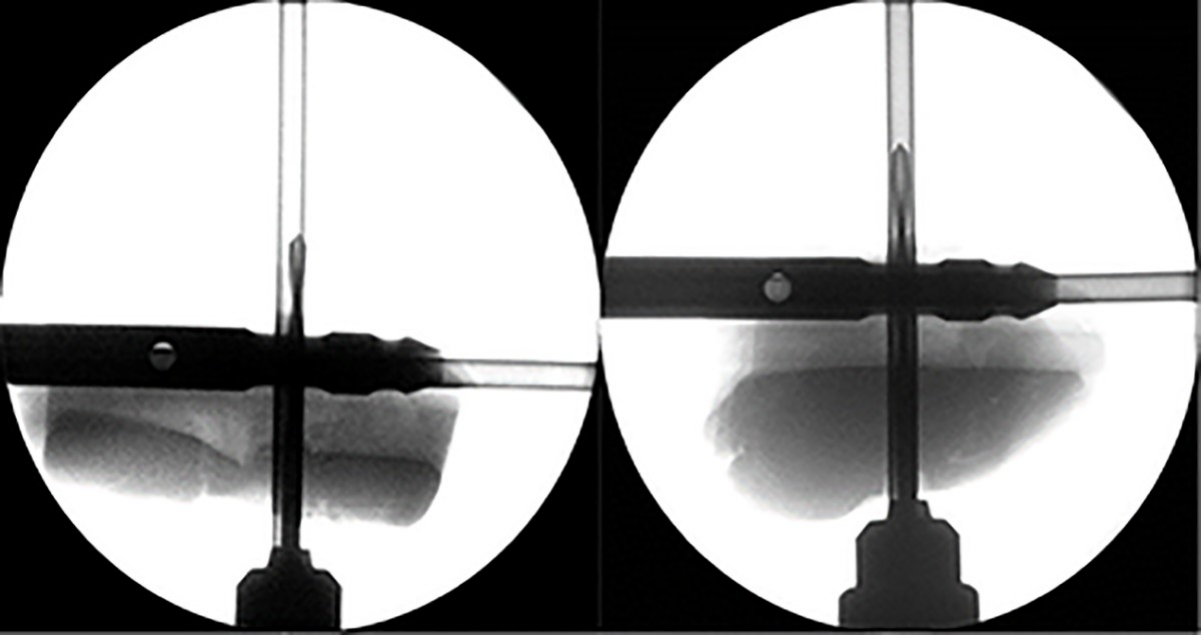
The C-arm radiographic images for evaluating the accuracy of the developed navigation system.

An in-vitro study with femur sawbones was conducted to evaluate the performance of the proposed system. A total of 30 trials were conducted by two novices and two experts, respectively. Fig 12 illustrates a photograph of the phantom experiment. The intersection point of the two line-laser beams indicates the insertion point of a drill bit. At the same time, the orientation information of the drill bit with respect to a distal hole is displayed on the screen in real time. In the phantom experiment, to verify whether the drill bit was successfully inserted into the distal hole of the nail, a C-arm image was acquired at every trial. Fig 13 shows that, using our system, the drill bit was inserted precisely into the distal hole of the nail. Among 120 trials for intramedullary nailing that were performed using this system, not a single case of failure was registered, thus demonstrating the potential of our system for intramedullary nailing.

For quantitative evaluation of performance of the system for intramedullary nailing, the incidence angle (θ) of the drill bit, the distance between the center line and the insertion point of the drill bit (A), and the distance between the center line and the exit point of the drill bit (B) were measured from the C-arm image. The results of this quantitative analysis of the system performance are shown in Table 2 and S1–S4 Tables. Comparing across Novice 1 and Novice 2, no significant differences were observed between the values of θ, between the values of |A|, between the values of |*B*|, and between the values of |B–A| (p-values = 0.691, 0.298, 0.290, and 0.740 > 0.05). In addition, the absolute angle of incidence for expert 1 was not significantly different from that for expert 2. The mean absolute angle of incidence for expert 1 was 3.35° (SD: ±2.19°), whereas that for expert 2 was 2.48° (SD: ±2.11°). In addition, no significant differences were observed between the mean values of |A|, |B|, and |B–A| for expert 1 and expert 2, respectively. The mean values of the θ, |A|, |B|, and |B–A| values for expert 1 were 3.35° (SD: ±2.19°), 1.99 mm (SD: ±1.65 mm), 2.42 mm (SD: ±1.84), and 0.64 mm (SD: ±0.42 mm), whereas those for expert 2 were 2.48° (SD: ±2.11°), 1.86 mm (SD: ±1.61 mm), 2.16 mm (SD: ±1.65 mm), and 0.48 mm (SD: ±0.40 mm), respectively.

**Table 2:** Results of the experiments for evaluating the developed system.

**Novice group**	**Intra-rater reliability; ICC**		**Inter-rater reliability; k**		**Reliability**
	**Novice 1**	**Novice 2**	**Novice 1**	**Novice 2**	
AV* of the incidence angle (θ, degrees)	2.47	2.02	1.47	1.52	r = 0.076, p = 0.691
**AV of the A (mm)**	1.84	2.47	1.57	1.69	r = 0.196, p = 0.298
**AV of the B (mm)**	2.22	2.75	1.72	1.80	r = 0.200, p = 0.290
**AV of the B-A (mm)**	0.48	0.38	0.28	0.29	r = 0.061, p = 0.748
**Expert group**	**Mean**		SD**		**Reliability**
	**Expert 1**	**Expert 2**	**Expert 1**	**Expert 2**	
**AV of the incidence angle (θ, degrees)**	3.35	2.48	2.19	2.11	r = 0.190, p = 0.314
**AV of the A (mm)**	1.99	1.86	1.65	1.61	r = 0.001, p = 0.996
**AV of the B (mm)**	2.42	2.16	1.84	1.65	r = 0.117, p = 0.539
**AV of the B-A (mm)**	0.64	0.48	0.42	0.40	r = 0.186, p = 0.324
**Novices vs. Experts**	**Mean**		**SD**		**Reliability**
	**Novices**	**Experts**	**Novices**	**Experts**	
**AV of the incidence angle (θ, degrees)**	2.25	2.92	1.10	1.66	r = 0.131, p = 0.489
**AV of the A (mm)**	2.16	1.92	1.26	1.15	r = 0.193, p = 0.308
**AV of the B (mm)**	2.49	2.29	1.37	1.22	r = 0.172, p = 0.363
**AV of the B-A (mm)**	0.43	0.56	0.21	0.32	r = 0.120, p = 0.526

* AV: absolute value

**SD: standard deviation

On comparison between the novice and expert groups, no significant differences between the θ values, the |A| values, the |B| values, and the |B–A| values were observed for the two groups. The mean values of θ, |A|, |B|, and |B–A| for the novice group were 2.25° (SD: ±1.10°), 2.16 mm (SD: ±1.26 mm), 2.49 mm (SD: ±1.37), and 0.43 mm (SD: ±0.21 mm), whereas those for the expert group were 2.92° (SD: ±1.66°), 1.92 mm (SD: ±1.15 mm), 2.29 mm (SD: ±1.22 mm), and 0.56 mm (SD: ±0.32 mm), respectively. These results demonstrate that, using the developed system, both the novice group and the expert group successfully performed precise intramedullary nailing with a high rate of success. This demonstrates the potential of our system for intramedullary nailing.

## Discussion

In this study, we developed a novel surgical navigation system with handle-integrated line-laser markers and inertial and magnetic sensor units for high-accuracy intramedullary nailing without radiation exposure. Using the system, an insertion point for distal locking was marked on skin, perpendicular to the center of the distal hole of the nail, allowing the drill bit to be inserted precisely into the distal hole of the nail. No failures were observed when intramedullary nailing was performed either by experts or novices.

**Fig 14:**
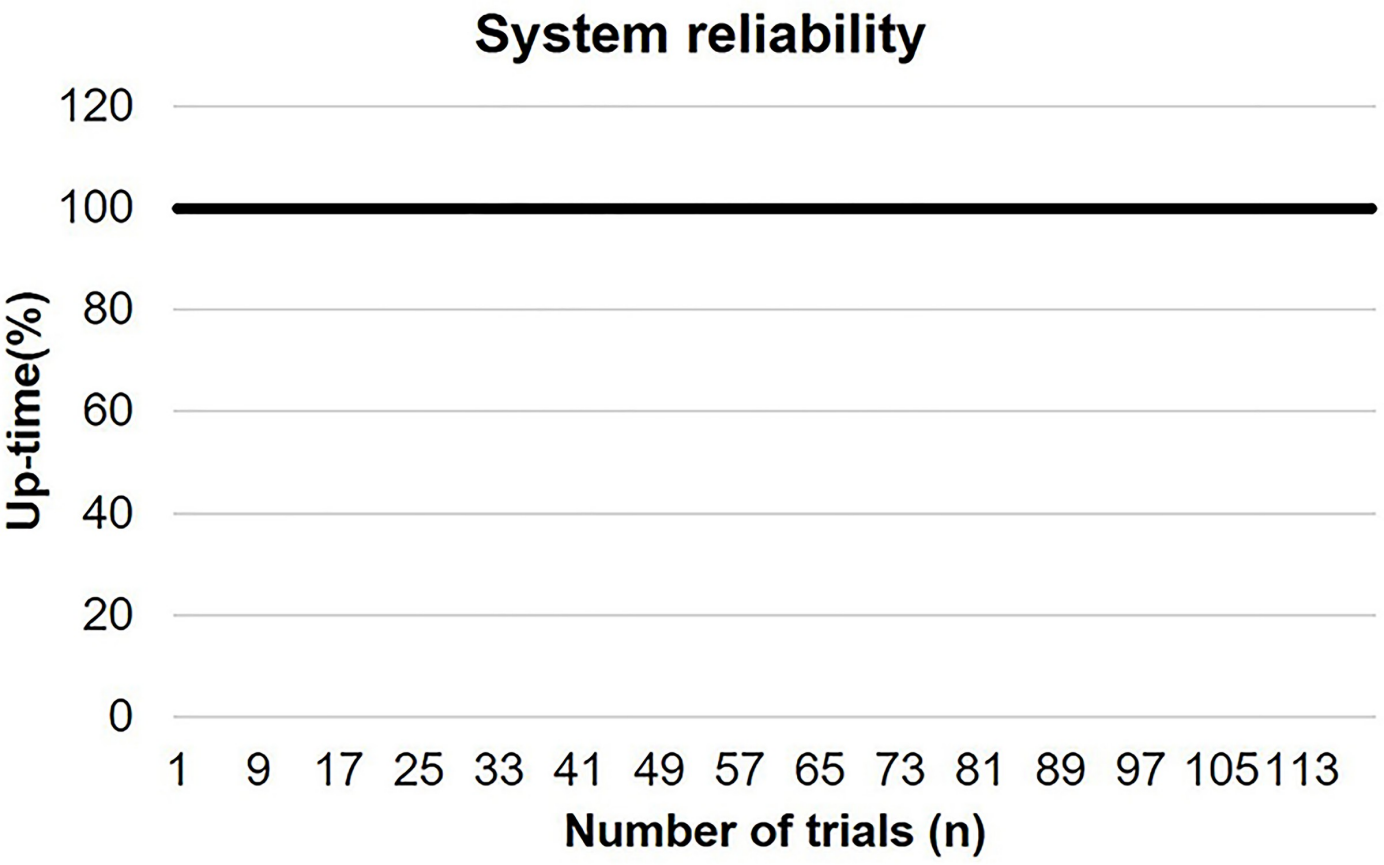
The normalized up-time in percentage during conducting a large number of experiment trials. The x-axis shows the number of trials.

For evaluation of the system reliability, we utilized the method presented in [17], which measures the system up-time and down-time distribution when devices were connected with a host computer wirelessly during a large number of experiment trials. Users who carried out the experiment needed to spend an average time of 10 minutes for distal locking operation using our system. While conducting 120 trials of experiment, the operation of our system with wireless connection to the host computer showed a distribution of 100% and 0% between up-time and down-time, respectively. The measured result is plotted in the Fig 14 shown below.

Even though the system that we developed has not so far exhibited any issues in the system reliability for surgical operations, our system may need to be upgraded for achieving further improved reliability. For that, a secondary Bluetooth module, which plays a role of the secondary slave channel, can be additionally implemented into our system. The secondary channel could be here utilized as a complementary channel for double-checking the transferred data by comparing the data received by the main channel and the secondary channel, and therefore it can improve the reliability in data transfer. However, this method may have several shortcomings such as an increase in the cost and power consumption of the system, and a prolonged pairing-time between master and slave channels. On the other hand, besides integrating the redundant module into our current system, an enhanced communication protocol can be also applied to our system in order to improve the system reliability. For instance, a master Bluetooth module repeatedly checks the header and check-sum of customized packets which consist of a customized header, angle data, and a check-sum, whenever the master receives the data. This protocol may thereby result in considerable improvements of the reliability in the data transfer. In addition, redundant data can be repeatedly transmitted two times as an alternative for improvement of the system reliability by increasing a Baud rate. If the Baud rate is increased, the Bluetooth communication system can transmit identical data two times whenever the data is transmitted. By doing so, the system reliability can be improved since the master can double-check the identical data received from the slave two times. Note that the Baud rate can be increased up to 2 Mbps in our developed system. Thus, the increased Baud rate of 2 Mbps was sufficient for real-time tracking of the system’s angle although the identical data were sent two times for the communication between surgical navigation tools.

Although the developed system needs to be calibrated for different types and lengths of nails before operation, it is important to note that this navigation system allows orthopedic surgeons to achieve intramedullary nailing in a precise and time-saving way, with few fluoroscopic image acquisitions. One aspect of challenges in intramedullary nailing that haven’t yet been addressed in this work is the compensation of the nail deformation induced during the nail insertion process [11, 18–20]. For developing a more complete and robust guidance solution, a proper compensation technique such as that presented in [19] has to be introduced and implemented as a part of the system. Such an approach may involve fluoroscopic imaging steps, but the number of required images can be strictly limited (e.g. two images used in [19].) Most importantly, the proposed system is simpler and cheaper compared with existing devices [3–6, 8–11]. The results of this study suggest that our developed system has great potential to serve as a novel tool for intramedullary nailing.

## Supporting information

S1 Table Data set of novice 1. This table shows several values of the experiment by novice 1.
(TIF)

S2 Table Data set of novice 2. This table shows several values of the experiment by novice 2.
(TIF)

S3 Table Data set of expert 1. This table shows several values of the experiment by expert 1.
(TIF)

S4 Table Data set of expert 2. This table shows several values of the experiment by expert 2.
(TIF)

S1 Movie Intramedullary Nailing phantom experiment and evaluation using C-arm radiographic. This video shows the process of the intramedullary nailing experiment using the system.
(WMV)
