# Supplementary figures and images for: A novel smart navigation system for intramedullary nailing in orthopedic surgery
Source: PLoS One. 2017 Apr 17;12(4):e0174407. doi: 10.1371/journal.pone.0174407 (PMC5393546; doi:10.1371/journal.pone.0174407)

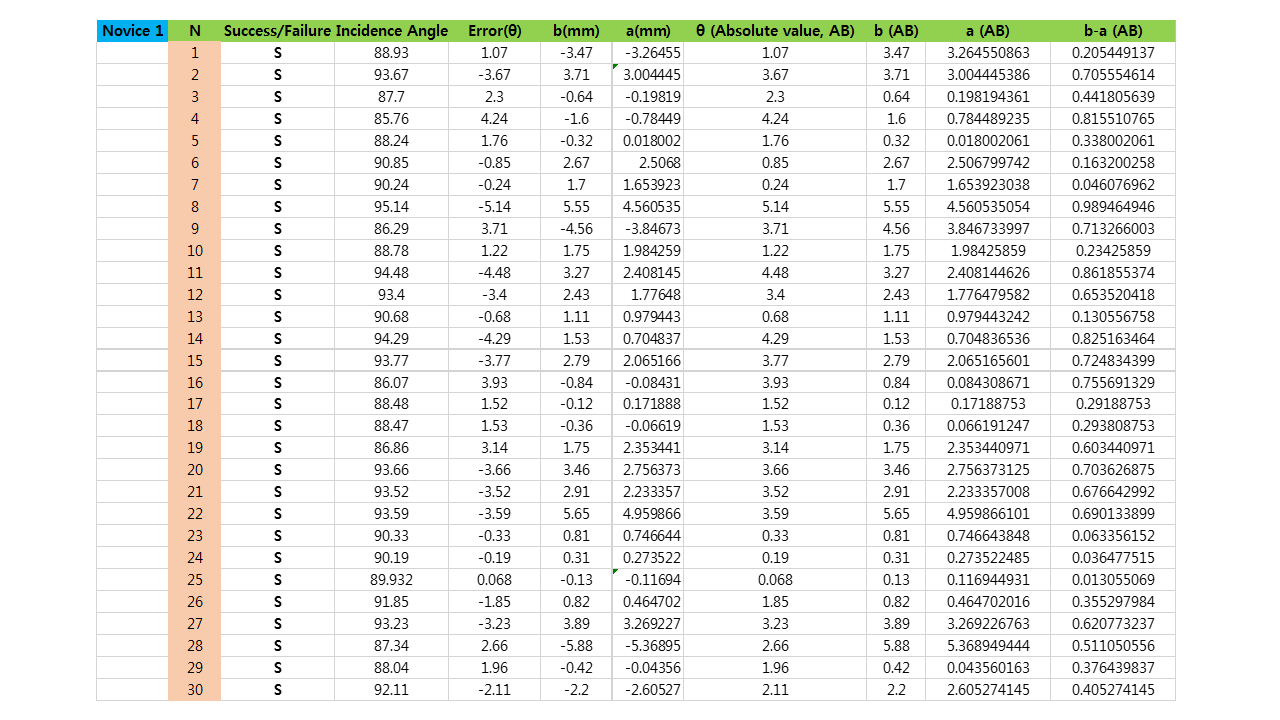

Supplement: S1 Table — This table shows several values of the experiment by novice 1. (TIF) [file pone.0174407.s001.TIF]

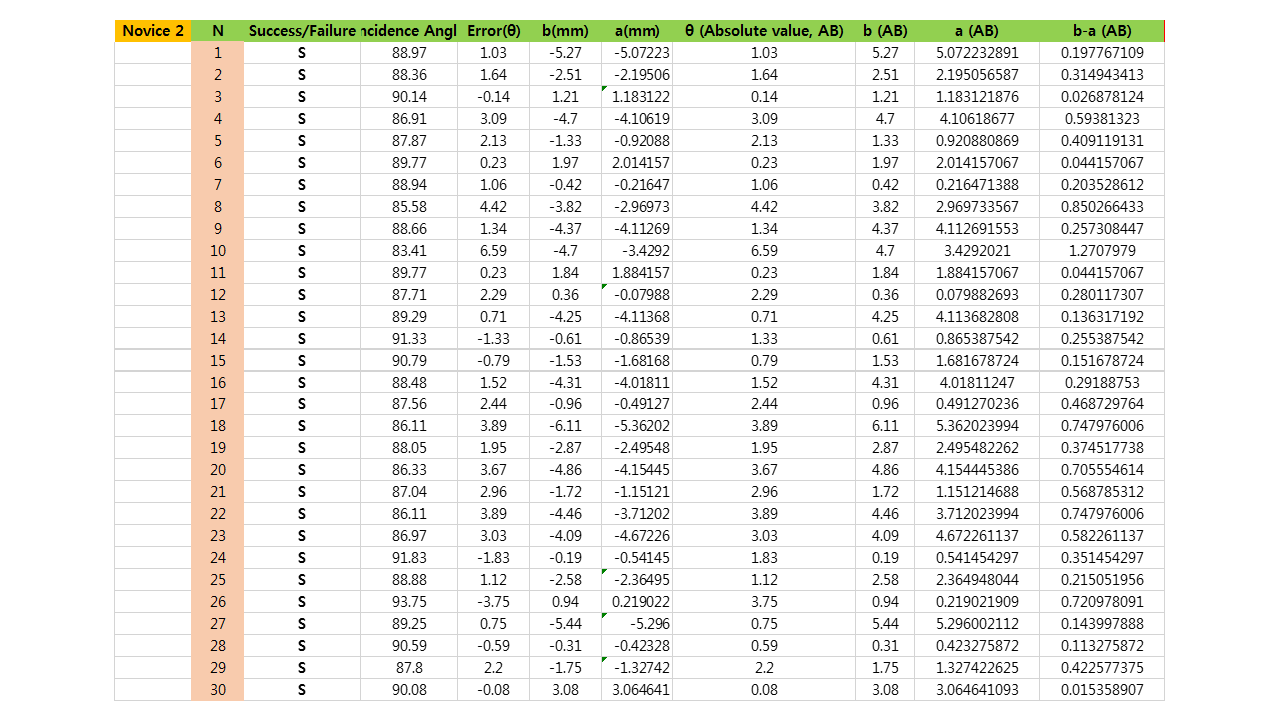

Supplement: S2 Table — This table shows several values of the experiment by novice 2. (TIF) [file pone.0174407.s002.TIF]

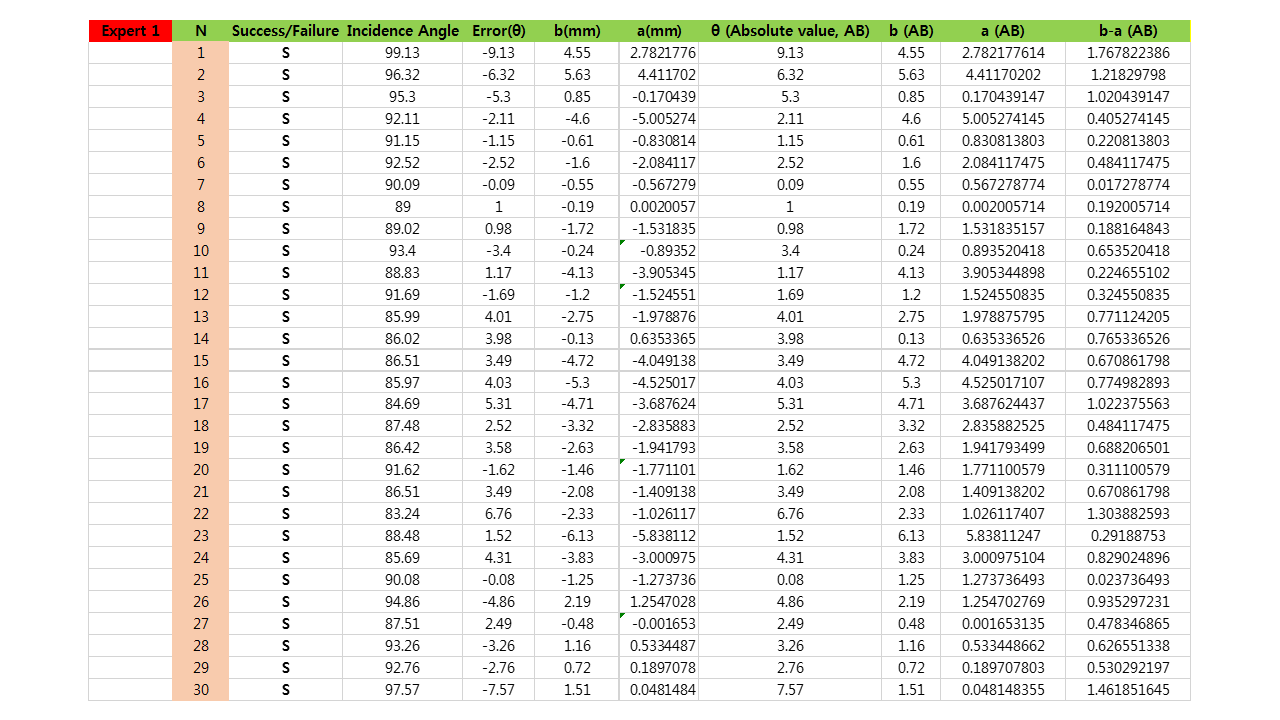

Supplement: S3 Table — This table shows several values of the experiment by expert 1. (TIF) [file pone.0174407.s003.TIF]

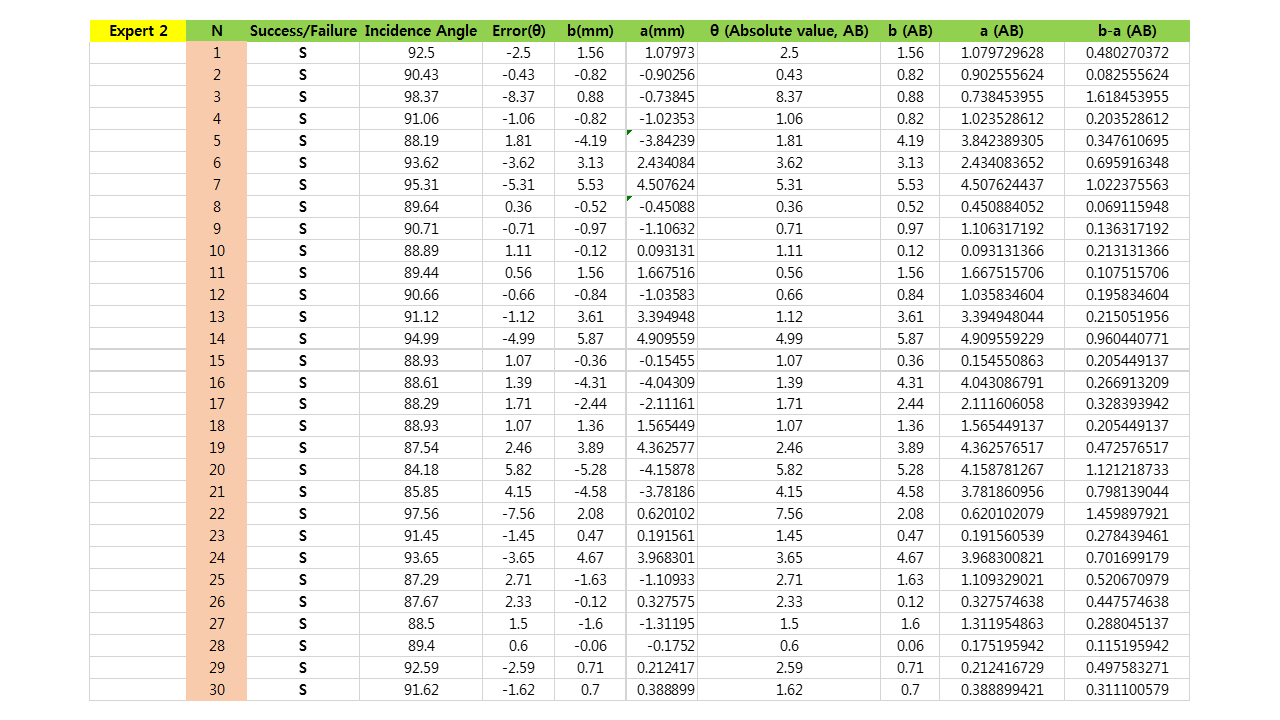

Supplement: S4 Table — This table shows several values of the experiment by expert 2. (TIF) [file pone.0174407.s004.TIF]
